# Two Japanese Pediatric Patients With Primary Ciliary Dyskinesia Caused by Loss-of-Function Variants in the CCNO gene

**DOI:** 10.7759/cureus.58854

**Published:** 2024-04-23

**Authors:** Yifei Xu, Koki Ueda, Tomoki Nishikido, Tsubasa Matsumoto, Kazuhiko Takeuchi

**Affiliations:** 1 Department of Otorhinolaryngology-Head and Neck Surgery, Mie University Graduate School of Medicine, Tsu, JPN; 2 Department of Pediatric Pulmonology and Allergy, Osaka Womenʼs and Childrenʼs Hospital, Izumi, JPN; 3 Department of Pediatric Infection and Immunology, Fukuoka Children’s Hospital, Fukuoka, JPN

**Keywords:** microtubular disorganization, ciliary ultrastructural defects, non cf bronchiectasis, chronic sinopulmonary disease, rare autosomal recessive disorder, reduced generation of multiple motile cilia (rgmc), ccno ( encoding cyclin o), primary ciliary dyskinesia (pcd)

## Abstract

Primary ciliary dyskinesia (PCD) is a rare congenital disorder caused by pathogenic variants of genes related to cilia. Here, we report two Japanese pediatric patients with PCD caused by pathogenic compound heterozygous variants in the cyclin O (*CCNO*) gene (Case 1, NM_021147.4:c.[262C>T];[781delC], p.[Gln88Ter];[Leu261fs]; Case 2, c.[262C>T];[c.248_252dupTGCCC], p.[Gln88Ter];[Gly85fs]). The clinical symptoms of the patients were varied. Neither of the patients had situs inversus. Transmission electron microscopy of the respiratory cilia from the nasal mucosa in Case 1 showed a remarkable reduction of cilia and the few residual cilia had central pair defects and microtubular disorganization.

## Introduction

Primary ciliary dyskinesia (PCD) is a congenital disorder caused by pathogenic variants of genes related to cilia. Most cases are inherited in an autosomal recessive manner, and more than 50 PCD causative genes have been reported [1]. Disease severity varies with the causative gene and variant [1,2]. PCD is characterized by persistent productive cough due to inefficient respiratory mucociliary clearance caused by ciliary beating disorders [1]. The varied symptoms of PCD include recurrent chronic airway infections, otitis media with effusion, infertility, and situs inversus [1].

Dynein axonemal heavy chain 5 (*DNAH5*), dynein axonemal intermediate chain 1 (*DNAI1*), and *DNAH11* are the most common causative PCD genes among most ethnicities, accounting for 15%-29%, 2%-10%, and 6%-9% of PCD patients, respectively, in European and American populations [1,2]. We reported recently that dynein regulatory complex subunit 1 (*DRC1*) and *DNAH5* are the most frequent causative genes in Japanese PCD patients [3-5]. Cases with variants in cyclin O (*CCNO*) are rare and account for less than 2% of PCD patients [1]. Here, we report for the first time two Japanese pediatric cases of PCD in whom CCNO was considered to be the causative gene by genetic testing.

## Case presentation

Case 1

**Figure 1:**
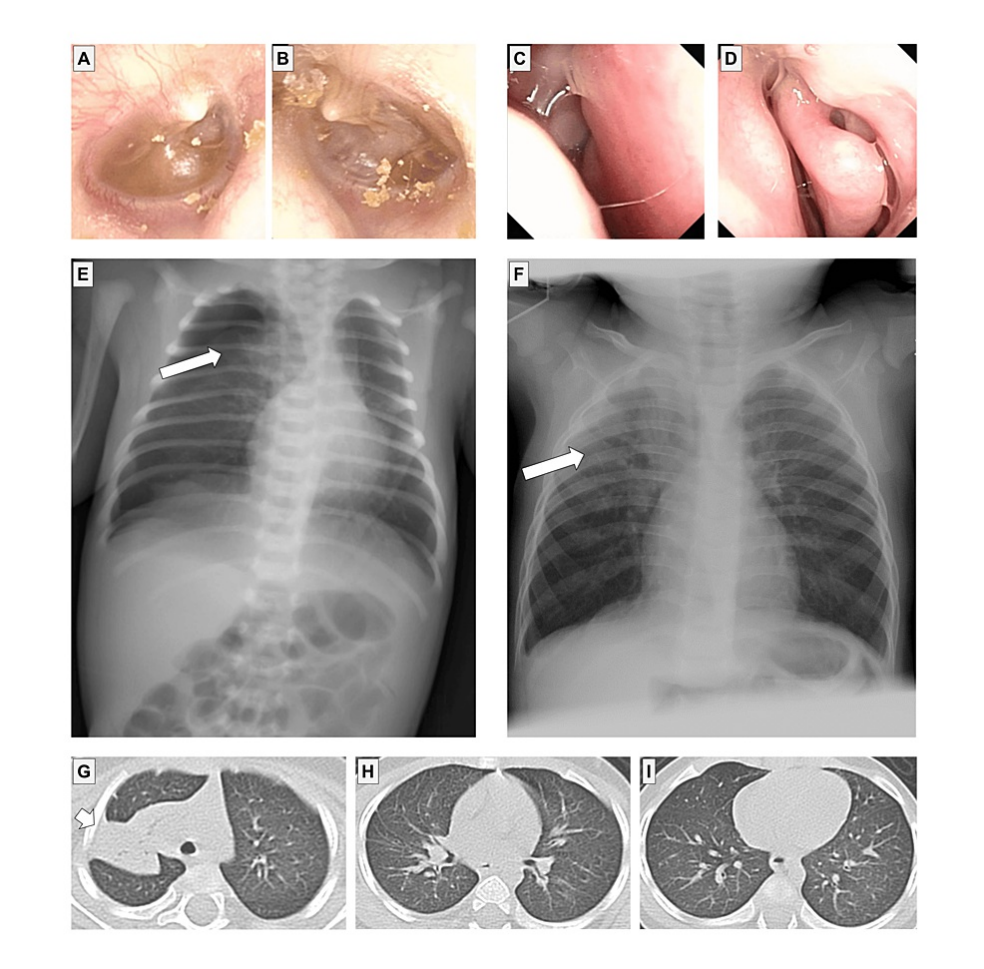
Imaging findings in Case 1. (A, B) Residual fluid and air-fluid levels in the bilateral middle ears. (C, D) Mucus secretion in the bilateral nasal cavities. (E) Chest X-rays immediately after birth and (F) at 10 months showed a shadow (arrows) in the right upper lobe. There was no situs inversus. (G) Chest computed tomography of the upper, (H) middle, and (I) lower lobes showed consolidation (arrow) in the right upper lobe.

**Figure 2:**
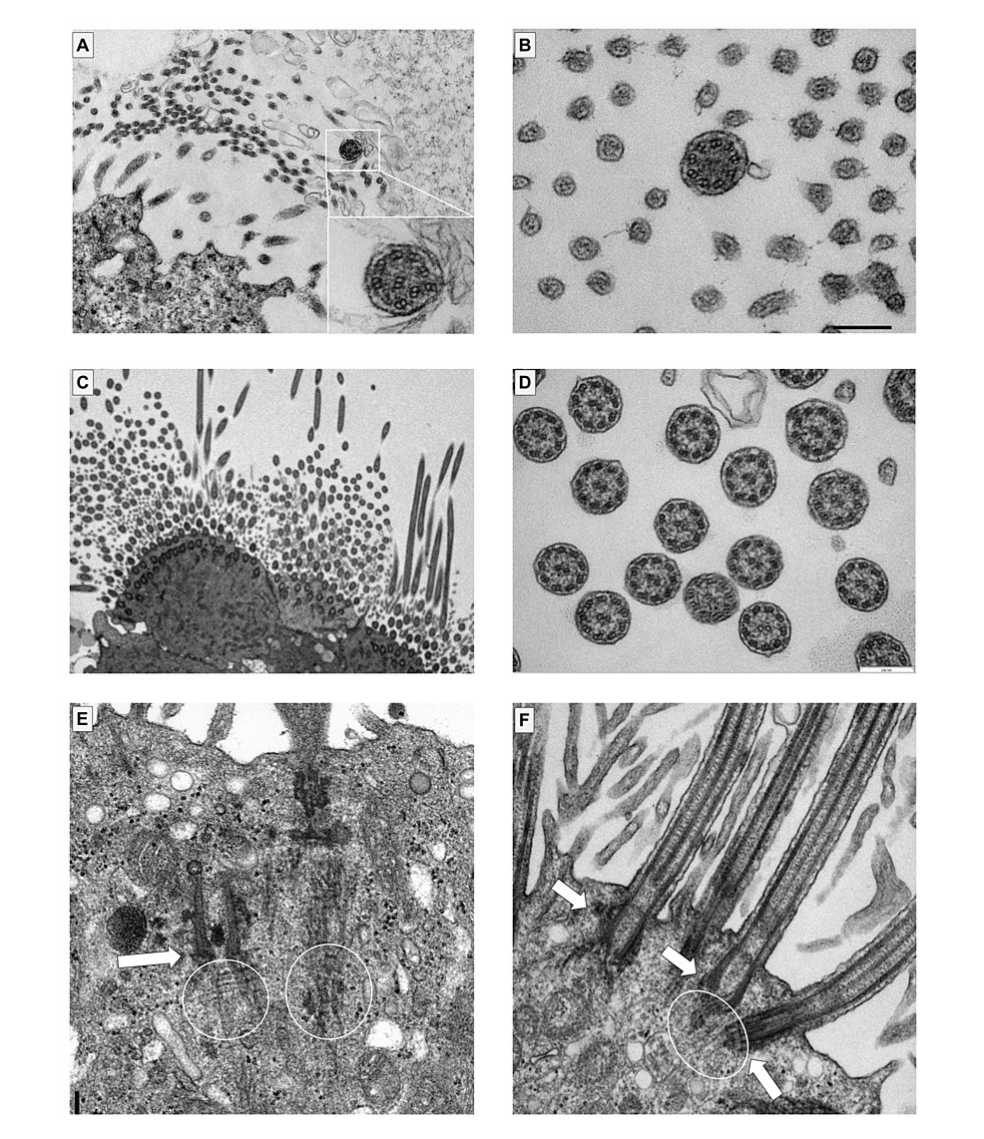
Transmission electron microscopy findings of a biopsy specimen from the nasal mucosa. (A) Longitudinal and (B) cross sections of Case 1 showing either complete absence or a remarkable reduction of cilia; (A) the residual cilium has microtubular disorganization; (B) the residual cilium lacks central pairs. (C, D) Cilia from healthy individuals (15-year-old female and 14-year-old male non-PCD controls) show the normal number of cilia; the cilia consist of one central pair of microtubules surrounded by nine pairs of well-arranged peripheral microtubules. (E) Nasal ciliated cells from Case 1. The basal bodies (arrows) and attached rootlets (circles) are mislocalized in the cytoplasm, far from the apical cell region. (F) Nasal ciliated cells from a healthy individual. The basal bodies attach to the rootlets and dock to the apical cell regions.

A 5-year-old boy was referred to our department for a persistent productive cough. He was born at 39 weeks. He had rhinorrhea and a productive cough immediately after birth and was admitted to the neonatal intensive care unit due to neonatal respiratory distress syndrome. He had persistent rhinosinusitis and refractory otitis media with effusion. He did not have situs inversus or a congenital heart defect. He did not have hydrocephalus. His parents and 9-year-old brother did not present with any symptoms of PCD. His PICADAR (PrImary CiliARy DyskinesiA Rule) score was 8 based on seven diagnostic questions to predict the likelihood of having PCD, indicating a 66% possibility of PCD. Rhonchi breath sounds were heard in both lungs with mild intercostal retraction. His heart rate was 120 bpm, and SpO_2_ was 98% on room air. *Haemophilus influenzae* (2+) was identified by a bronchoscopic culture. A nasal nitride oxide (nNO) test was not performed. He had residual fluid in the bilateral middle ears (Figure 1A, 1B). Mucus secretions were observed in the bilateral nasal cavities on nasal endoscopy (Figure 1C, 1D). Chest X-rays immediately after birth and at 10 months had shown a shadow in the right upper lobe (Figure 1E, 1F). Chest computed tomography showed consolidation in the right upper lobe (Figure 1G-1I). Transmission electron microscopy of respiratory cilia from the nasal mucosa showed either a complete absence or severely decreased numbers of cilia (Figure 2).

**Figure 3:**
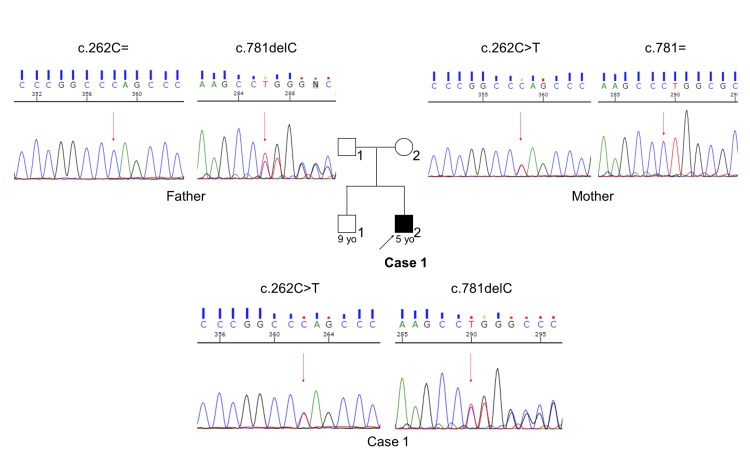
Family pedigree of Case 1 with the results of Sanger sequencing. Compound heterozygous variants in *CCNO* are shown.

Exome sequencing performed in 32 known PCD genes [4] identified two heterozygous variants in *CCNO* (NM_021147.4:c.[262C>T];[781delC], p.[Gln88Ter];[Leu261fs]); causative pathogenic variants in other PCD genes could not be found. Both variants were predicted to be loss-of-function mutations, and neither has been reported previously. Sanger sequencing of Case 1 and his parents confirmed the presence of compound heterozygous variants (Figure 3). His mother carried the former variant and his father carried the latter variant.

Case 2

**Figure 4:**
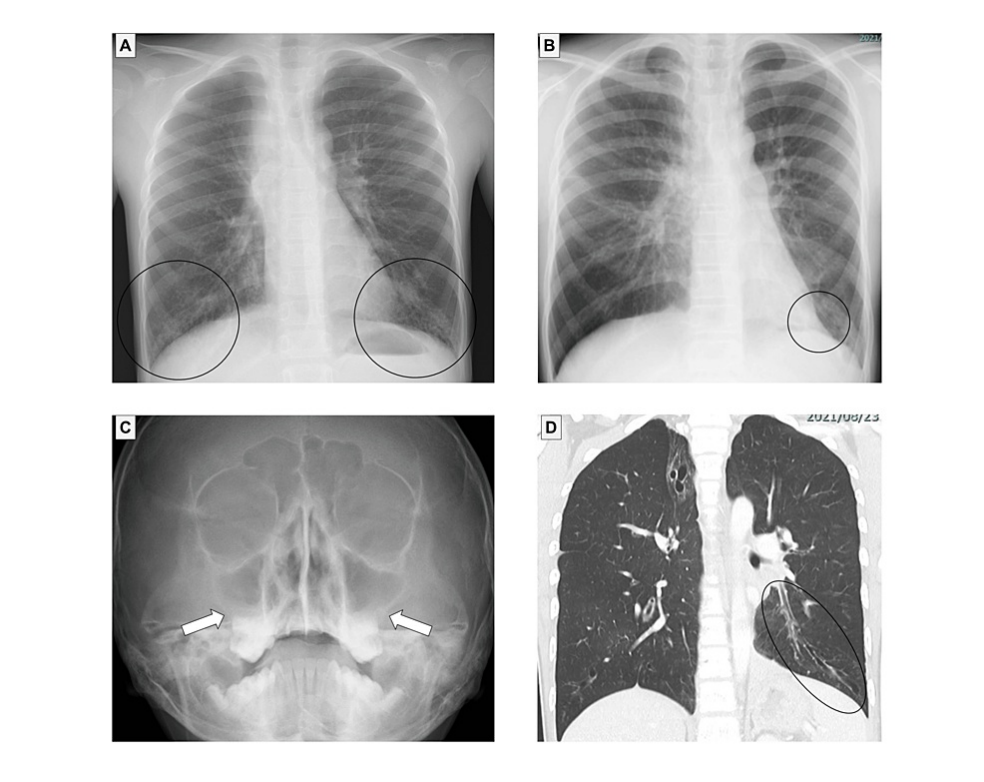
Imaging findings in Case 2. (A) Chest X-ray at 5 years of age showed infiltrative shadows (circles) in the bilateral lower lung. (B) Chest X-ray showed atelectasis in the left lower lobe (circle). (C) Nasal X-ray at 13 years of age showed thickened mucosa of the bilateral maxillary sinuses (arrows). (D) Chest computed tomography at 13 years of age showed bronchiectasis (circle).

The patient was a 13-year-old boy with a persistent wet cough whose blood samples were sent to our department for genetic testing. He was born at full term and was admitted to a neonatal intensive care unit due to pneumonia on the second day after birth. He had been suffering from refractory pneumonia (Figure 4A), atelectasis (Figure 4B) and chronic rhinosinusitis (Figure 4C). He was hospitalized due to bacterial pneumonia at 13 years of age, and bronchiectasis was noted at that time (Figure 4D). He did not have situs inversus or a congenital heart defect. He did not have hydrocephalus. His parents and 12-year-old sister did not present with the same symptoms. His PICADAR score was 7, indicating a 44% possibility of PCD. Breath sounds were diminished in the left lung and coarse crackles were heard in the right lower lung. His body temperature was 38.5°C, SpO_2_ 88% (room air), heart rate was 120 bpm, and respiratory rate was 26 breaths/min. Forced vital capacity (FVC) was 3.57 L (94.2%), Forced expiratory volume in the first second (FEV_1_) was 2.64 L (77.0%), FEV_1_% was 73.95% at the time of one-year follow-up. *Streptococcus pneumoniae* (PSSP) (1+), *Neisseria* species (small amount), and *Haemophilus parainfluenzae* (small amount) were identified by sputum culture. A nasal nitride oxide (nNO) test was not conducted, and exhaled nitric oxide was 8ppb.

**Figure 5:**
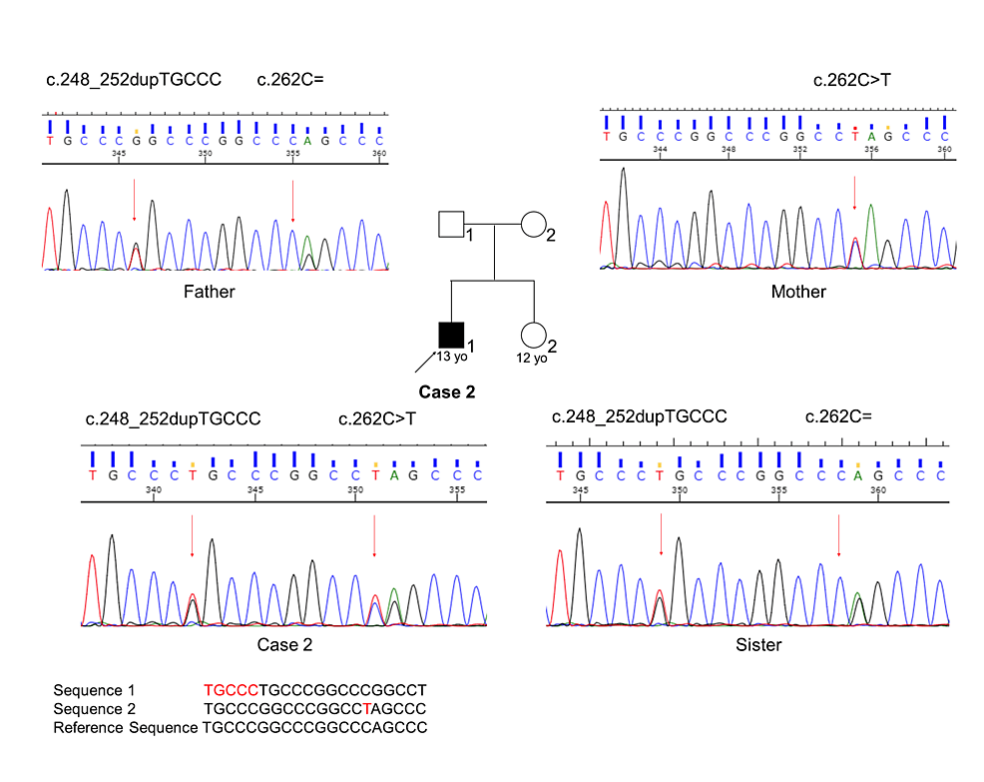
Family pedigree of Case 2 with the results of Sanger sequencing. Compound heterozygous variants in *CCNO* are shown.

Exome sequencing performed in 32 known PCD genes [4] identified two heterozygous variants in *CCNO* (NM_021147.4:c.[262C>T];[c.248_252dupTGCCC], p.[Gln88Ter];[Gly85fs]); causative pathogenic variants in other PCD genes could not be found. Sanger sequencing confirmed that the mother carried the former variant, while the father carried the latter one (Figure 5).

Findings from both cases are summarised in Table 1.

**Table 1:** Clinical characteristics and examination findings of patients. FCV: Forced vital capacity; FEV_1_:Forced expiratory volume in the first second

	Case 1	Case 2
Sex	Male	Male
Age at diagnosis	5	13
FVC	NA	3.57 L (94.2%)
FEV_1_	NA	2.64 L (77.0%)
FEV_1_%	NA	73.95%
Sputum culture	*Haemophilus influenzae* (2+)	*Streptococcus pneumoniae* (PSSP) (1+), *Neisseria* species (small amount), *Haemophilus parainfluenzae* (small amount).
Persistent wet cough	Yes	Yes
Born	Full-term	Full-term
Neonatal respiratory distress	Yes	Yes
Admission to a neonatal intensive care unit	Yes	Yes
Respiratory symptoms in the neonatal period	Rhinorrhea, productive cough	Pneumonia, atelectasis
Persistent rhinosinusitis	Yes	Yes
Refractory otitis media with effusion	Yes	No
Bronchiectasis	No	Yes
Atelectasis	No	Yes
Situs inversus	No	No
Congenital heart defect	No	No
Hydrocephalus	No	No
Complementary DNA Change (Protein Change)	c.262C>T (p.Gln88Ter), maternal	c.262C>T (p.Gln88Ter), maternal
	c.781delC (p.Leu261fs), paternal	c.248_252dupTGCCC (p. Gly85fs), paternal
Transmission electron microscopy	either a complete absence or severely decreased numbers of cilia	NA

## Discussion

To our knowledge, 55 PCD cases caused by CCNO worldwide have been published in the literature (Supplementary Material 1). This is the first report of PCD in Japanese patients caused by pathogenic variants in *CCNO*. Wallmeier et al. first reported disease-causing *CCNO* variants in 16 patients with chronic destructive lung disease due to insufficient airway clearance and identified a homozygous variant (c.248_252dupTGCCC, p.Gly85Cysfs*10) in a large consanguineous Kuwaiti family [6], which is the same variant found in Case 2 in the present report. The same variant was also reported recently in a Chinese female PCD patient [7]. In addition, we identified two novel variants, the nonsense mutation c.262C>T (rs1272978797) in Cases 1 and 2, and the frameshift deletion mutation c.781delC in Case 1 (Supplementary Material 2). Neither of these variants has been reported in PCD patients.

CCNO is a cyclin-like protein and has been predicted to function in the cell cycle and transcriptional control. It plays a critical regulatory role in deuterosome-mediated centriole amplification during multiciliogenesis [8]. Multiciliated cells have specific procentriole nucleation centers called deuterosomes, where the massive amplification of centrioles is achieved [9]. During cilia biogenesis, centrioles amplify and dock to the apical cell membrane to form basal bodies and serve as a platform and rootlet for the outgrowth of ciliary axonemes [10]. Ccno-deficient multiciliated cells fail to generate sufficient numbers of functional deuterosomes, resulting in a marked reduction and maturation defects of centrioles. The centrioles fail to localize correctly during their transition to basal bodies. As a result, *CCNO*-deficient cells show reduced numbers of motile cilia on their apical surface, leading to mucociliary clearance defects [8,11].

Consistent with a previous report [6], transmission electron microscopy in Case 1 in the present report showed that the basal bodies and rootlets were mislocalized within the cytoplasm of nasal ciliated cells, not at the apical cell region, suggesting a defect in centriole generation and basal body migration. Compared with approximately 200 cilia per ciliated cell in healthy controls, cases with CCNO defects have only one or two cilia per cell [6,12]. This CCNO deficiency may lead to ultrastructural defects of axonemes [8]. Case 1 had central pair defects and microtubular disorganization. These ciliary ultrastructural defects might be caused primarily by CCNO deficiency or as a result of secondary defects due to increased susceptibility to inflammation or infection.

Our two pediatric cases presented with severe respiratory symptoms early in life. *CCNO* cases reportedly show rapid deterioration in lung function, and *CCNO* pediatric cases are more likely to have bronchiectasis, with an incidence of 89% [13]. In contrast, the incidence of bronchiectasis among other pediatric PCD cohorts has been reported as 50%-75% [14] and 38% [15]. *CCNO* cases also have a higher incidence of neonatal respiratory distress syndrome [13-15].

*CCNO* deficiency causes defective ciliary function of ependymal multi-ciliated cells, reducing the intraventricular transport of cerebrospinal fluid and leading to hydrocephalus. The clinical symptoms in humans and mice appear to vary; hydrocephalus is not common in humans and mice do not show obvious respiratory problems [8]. *Ccno* deletion causes 57% of the mutant mice to develop hydrocephalus [8]; a systematic analysis of *CCNO* cases revealed that hydrocephalus occurs in 10% of human patients [13]. Our two cases did not have hydrocephalus. They also did not have situs inversus, probably because the axonemal motor proteins of the few residual cilia are expressed correctly leading to intact ciliary beating during organ asymmetry at the node in early-somite stage embryos [6].

## Conclusions

We encountered two Japanese pediatric patients with PCD caused by pathogenic variants in *CCNO*. The clinical symptoms of the patients were varied, and neither of the patients had situs inversus. Case 1 in the present report showed a remarkable reduction of cilia and an abnormal ciliary ultrastructure of mislocalized basal bodies and rootlets by transmission electron microscopy, suggesting a defect in centriole generation and basal body migration. We also observed and first reported ciliary ultrastructural defects of axonemes in the *CCNO* case. In addition, we identified two novel variants, namely *CCNO* NM_021147.4:c.262C>T (p.Gln88Ter) and c.781delC (p.Leu261fs).

## Figures and Tables

**Figure 6:**
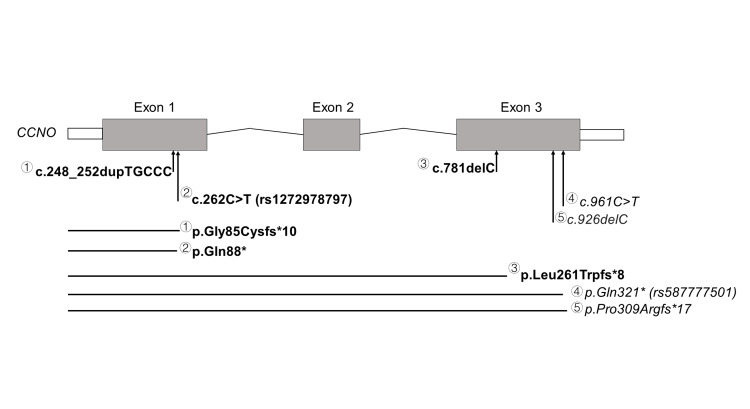
Schematic of CCNO gene and predicted consequences of the variants at the protein level. All the variants are marked with arrows. All the variants are classical loss-of-function mutations. Disease-causing variants reported in the literature (in italics) [6] are also shown in the figure.

## Appendices

Supplementary material 1

Table showing CCNO gene mutation studies.

**Table 2:** CCNO gene mutation studies.

Study	Number of CCNO cases	Pathogenic variants in CCNO	Clinical phenotype	Ciliary ultrastructure and function
Wallmeier et al. (2014) [6]	16	c.[248_252dupTGCCC];[248_252dupTGCCC], p.[Gly85Cysfs*10]; [Gly85Cysfs*10] in four patients from a consanguineous Kuwaiti family and in one individual patient. c.[248_252dupTGCCC];[481_482delCT], p.[Gly85Cysfs*10]; [Leu161Glyfs*72] in one patient. c.[263_267dupAGCCC];[263_267dupAGCCC], p.[Val90Serfs*5]; [Val90Serfs*5] in two patients of one family. c.[258_262dupGGCCC];[258_262dupGGCCC], p.[Gln88Argfs*7]; [Gln88Argfs*7] in five patients of three families. c.[716A>G];[716A>G], p.[His239Arg]; [His239Arg] in one patient. c.[961C>T];[961C>T], p.[Gln321*]; [Gln321*] in one patient. c.[926delC];[926delC], p.[Pro309Argfs*17]; [Pro309Argfs*17] in one patient.	All had chronic destructive lung disease, bronchiectasis in early childhood. Twelve newborns had respiratory distress. Two patients had terminal respiratory failure and lung transplantation at 34 y.o. One female had infertility. Situs solitus. Low nasal nitric oxide (nNO) in 11 patients.	Reduction in the number of multiple motile cilia, residual cilia were motile and without obvious beating defects.
Casey et al. (2015) [16]	2	c.[258_262dupGGCCC];[258_262dupGGCCC], p.[Gln88Argfs*7]; [Gln88Argfs*7] in two siblings from an Irish Traveller family.	All had recurrent lower respiratory tract infections. No recurrent sinusitis. The elder sibling had recurrent otitis media. Situs solitus. nNO at 30-50 ppb.	Ciliary aplasia.
Amirav et al. (2016) [13]	15	c.165delC, p.Gly56Alafs*38; c.481_482delCT, p.Leu161Glyfs*73; c.638T>C, p.Leu213Pro; c.262_263dupGGCCC, p.Gln88Argfs*8; c.258_262dupGGCCCC; p.Gln88Argfs*8 in 15 patients of 10 Israel families.	Neonatal respiratory distress syndrome (11/14), recurrent otitis media (10/15), sinusitis (13/14), bronchiectasis (13/14). One patient had lung transplantation at 43 y.o. One female had in vitro fertilization assistance. One patient had hydrocephalus. Situs solitus. nNO of 11 patients ranges at 7-76 nL/min, three patients at a normal range of 99-256 nL/min.	Reduction in the number of respiratory cilia.
Guo et al. (2017) [17]	2	c.[248_249insGCCCG];[248_249insGCCCG], p.[Q88Rfs*8];[Q88Rfs*8] from one Chinese family.	All had bronchiectasis and rhinosinusitis. Situs solitus. nNO 23.6 ppb and 3.3 ppb.	NA
Shen et al. (2019) [18]	1	c.[848T>C];[262_263insGGCCCGGCCC], p.[L283P];[Q88Rfs*51]	Severe pneumonia, bronchiectasis, nasosinusitis, pneumothorax.	NA
Emiralioğlu et al. (2020) [19]	4	c.[564_567+1delinsGCGATGCAAGCGATGC AAGCGATGCAAGCGATGA];[564_567+1 DelinsGCGATGCAAGCGATGCAAGCGATG CAAGCGATGA], splicing-mut. of two patients from two families. c.[263_267dupAGCCC];[263_267dupAGCCC], p.[Val90Serfs*6];[Val90Serfs*6] of two patients from two families.	All had respiratory distress. Situs solitus. nNO range at 5-18 ppb.	Hypokinetic cilia by high-speed video microscopy.
Guo et al. (2020) [15]	1	c.[615delC];[253_262dupGGCCCGGCCC], p.[Cys205Trpfs∗64]; [Gln88Argfs∗51].	nNO 15.1 nL/min.	No cilia.
Guan et al. (2021) [20]	3	c.[267_c.268insAGCCC], p.[V90Sfs∗6]. c.[262_c.263insGGCCC];[262_c.263insGGCCC], p.[Q88Rfs∗8];[Q88Rfs∗8]. c.[262_c.263insGGCCCGGCCC];[262_c.263ins GGCCCGGCCC], p.[Q88Rfs∗51];[Q88Rfs∗51]	Two patients had neonatal respiratory distress. All patients had telectasis and one had bronchiectasis. Situs solitus. nNO range at 17-56 ppb.	Oligocilia.
Henriques et al. (2021) [21]	3	c.[253_257GGCCC];[263_267dup], p.[Gln88fs];[Val90fs]. c.[263_267dup];[263_267dup], p.[Val90fs];[Val90fs] in two patients from two families.	Early-onset respiratory symptoms. Two patients had neonatal respiratory distress, two had atelectasis and lobar collapse, one had lobectomy. One patient had mild ventriculomegaly. Situs solitus. nNO range at 11-137 ppb.	Severely decreased numbers of cilia. Normal ultrastructure and uncoordinated beat pattern in the residual cilia.
Ma et al. (2021) [22]	1	c.[262_263insGGCCC];[262_263insGGCCC], p.[Gln88Argfs*8];[Gln88Argfs*8].	Chronic airway infection, infertility.	Decreased numbers of multiciliated cells in the epithelium of the oviduct.
Zhang et al. (2022) [7]	1	c.[303C>A];[248_252dup], p.[Tyr101*];[Gly85Cysfs*10]	Recurrent wet cough since birth and occasional dyspnea. Bronchiectasis.	Shortened and reduction in the number of respiratory cilia.
Wang et al. (2023) [23]	1	c.[263_267dupAGCCC];[258_262dupGGCCC], p.[Val90Serfs*5];[Gln88Argfs*7]	Recurrent respiratory infections. Dyspnea at birth. Had slightly widened lateral ventricle. Bronchiectasis.	No cilia.
Gong et al. (2023) [24]	1	c.[323del];[323del], p.[F108Sfs*21];[F108Sfs*21]	Recurrent wet cough. Otitis media. Slow growth in height since childhood. Short Stature. Situs solitus.	NA
Petrarca et al. (2023) [25]	1	c.[258_262dup];[258_262dup], p.[Gln88Argfs*8];[Gln88Argfs*8]	Neonatal respiratory distress. Increased volume of choroid plexuses and lateral ventricles. Acute respiratory distress.	Inconclusive.
Celiksoy et al. (2023) [26]	1	c.[493delC];[493delC], p.[Leu165Serfs*33];[Leu165Serfs*33]	Recurrent respiratory infections since infancy. Neonatal respiratory distress.	The cilia were short and had minimal or no movement.
Alhalabi et al. (2024) [27]	2	c.[258_262dup];[258_262dup], p.[Gln88Argfs*8];[Gln88Argfs*8] in two Indian siblings.	Recurrent respiratory infections since infancy. Obstructive ventilatory dysfunction. One needed lung transplantation at 17 y.o. Situs solitus. nNO at 63.3 ppb and 88.3 ppb.	NA

Supplementary material 2
